# Publisher Correction: Novel pleiotropic risk loci for melanoma and nevus density implicate multiple biological pathways

**DOI:** 10.1038/s41467-018-08078-w

**Published:** 2019-01-14

**Authors:** David L. Duffy, Gu Zhu, Xin Li, Marianna Sanna, Mark M. Iles, Leonie C. Jacobs, David M. Evans, Seyhan Yazar, Jonathan Beesley, Matthew H. Law, Peter Kraft, Alessia Visconti, John C. Taylor, Fan Liu, Margaret J. Wright, Anjali K. Henders, Lisa Bowdler, Dan Glass, M. Arfan Ikram, André G. Uitterlinden, Pamela A. Madden, Andrew C. Heath, Elliot C. Nelson, Adele C. Green, Stephen Chanock, Jennifer H. Barrett, Matthew A. Brown, Nicholas K. Hayward, Stuart MacGregor, Richard A. Sturm, Alex W. Hewitt, Melanoma GWAS Consortium, Jeffrey E. Lee, Myriam Brossard, Eric K. Moses, Fengju Song, Rajiv Kumar, Douglas F. Easton, Paul D. P. Pharoah, Anthony J. Swerdlow, Katerina P. Kypreou, Mark Harland, Juliette Randerson-Moor, Lars A. Akslen, Per A. Andresen, Marie-Françoise Avril, Esther Azizi, Giovanna Bianchi Scarrà, Kevin M. Brown, Tadeusz Dębniak, David E. Elder, Shenying Fang, Eitan Friedman, Pilar Galan, Paola Ghiorzo, Elizabeth M. Gillanders, Alisa M. Goldstein, Nelleke A. Gruis, Johan Hansson, Per Helsing, Marko Hočevar, Veronica Höiom, Christian Ingvar, Peter A. Kanetsky, Wei V. Chen, Maria Teresa Landi, Julie Lang, G. Mark Lathrop, Jan Lubiński, Rona M. Mackie, Graham J. Mann, Anders Molven, Srdjan Novaković, Håkan Olsson, Susana Puig, Joan Anton Puig-Butille, Graham L. Radford-Smith, Nienke van der Stoep, Remco van Doorn, David C. Whiteman, Jamie E. Craig, Dirk Schadendorf, Lisa A. Simms, Kathryn P. Burdon, Dale R. Nyholt, Karen A. Pooley, Nicholas Orr, Alexander J. Stratigos, Anne E. Cust, Sarah V. Ward, Hans-Joachim Schulze, Alison M. Dunning, Florence Demenais, Christopher I. Amos, Manfred Kayser, David J. Hunter, Julia A. Newton Bishop, Timothy D. Spector, Grant W. Montgomery, David A. Mackey, George Davey Smith, Tamar E. Nijsten, D. Timothy Bishop, Veronique Bataille, Mario Falchi, Jiali Han, Nicholas G. Martin

**Affiliations:** 1 QIMR Berghofer Medical Research Institute, Brisbane, Australia; 2 Department of Epidemiology, Richard M. Fairbanks School of Public Health, Melvin and Bren Simon Cancer Center, Indiana University, Indianapolis, IN 63110 USA; 3 Department of Twin Research & Genetic Epidemiology, St Thomas Hospital Campus, Kings College, London, UK; 4 Section of Epidemiology and Biostatistics, Leeds Institute of Cancer and Pathology, University of Leeds, Leeds, UK; 5 Department of Dermatology, Erasmus MC, University Medical Centre Rotterdam, Rotterdam, The Netherlands; 6 MRC Integrative Epidemiology Unit, University of Bristol, Bristol, UK; 7 University of Queensland Diamantina Institute, Translational Research Institute, Brisbane, Australia; 8 Centre for Ophthalmology and Vision Science, University of Western Australia and the Lions Eye Institute, Perth, Australia; 9 Department of Epidemiology, Harvard T.H. Chan School of Public Health, Boston, 02115 MA USA; 10 Department of Genetic Identification, Erasmus MC, University Medical Centre Rotterdam, Rotterdam, The Netherlands; 11 Department of Epidemiology, Erasmus MC, Rotterdam, Netherlands; 12 Department of Internal Medicine, Erasmus MC, Rotterdam, Netherlands; 13 Department of Psychiatry, Washington University School of Medicine, St. Louis, MO 63110 USA; 14 Molecular Oncology Group, CRUK Manchester Institute, University of Manchester, Manchester, UK; 15 Division of Cancer Epidemiology and Genetics, National Cancer Institute, Bethesda, MD USA; 16 Dermatology Research Centre, University of Queensland Diamantina Institute, Translational Research Institute, Brisbane, Australia; 17 Present Address: Institute for Molecular Bioscience, The University of Queensland, Brisbane, Australia; 18 Department of Surgical Oncology, The University of Texas MD Anderson Cancer Center, Houston, TX USA; 19 Institut National de la Santé et de la Recherche Médicale (INSERM), UMR-946, Genetic Variation and Human Diseases Unit, Paris, France; 20 Centre for Genetic Origins of Health and Disease, Faculty of Medicine, Dentistry and Health Sciences, The University of Western Australia, Western Australia, Australia; 21 Departments of Epidemiology and Biostatistics, Key Laboratory of Cancer Prevention and Therapy, National Clinical Research Center of Cancer, Tianjin Medical University Cancer Institute and Hospital, Tianjin, 300060 P. R. China; 22 Division of Molecular Genetic Epidemiology, German Cancer Research Center, Im Neuenheimer Feld 580, Heidelberg, Germany; 23 Centre for Cancer Genetic Epidemiology, Department of Public Health and Primary Care, University of Cambridge, Cambridge, UK; 24 Centre for Cancer Genetic Epidemiology, Department of Oncology, University of Cambridge, Cambridge, UK; 25 Division of Genetics and Epidemiology, The Institute of Cancer Research, London, UK; 26 Department of Dermatology, University of Athens School of Medicine, Andreas Sygros Hospital, Athens, Greece; 27 Section of Epidemiology and Biostatistics, Leeds Institute of Cancer and Pathology, University of Leeds, Leeds, UK; 28 Centre for Cancer Biomarkers CCBIO, Department of Clinical Medicine, University of Bergen, Bergen, Norway; 29 Department of Pathology, Molecular Pathology, Oslo University Hospital, Rikshospitalet, Oslo, Norway; 30 Assistance Publique–Hôpitaux de Paris, Hôpital Cochin, Service de Dermatologie, Université Paris Descartes, Paris, France; 31 Department of Dermatology, Sheba Medical Center, Tel Hashomer, Sackler Faculty of Medicine, Tel Aviv, Israel; 32 Department of Internal Medicine and Medical Specialities, University of Genoa, Genoa, Italy; 33 Division of Cancer Epidemiology and Genetics, National Cancer Institute, National Institutes of Health, Bethesda, MD USA; 34 International Hereditary Cancer Center, Pomeranian Medical University, Czechs, Poland; 35 Department of Pathology and Laboratory Medicine, Perelman School of Medicine at the University of Pennsylvania, Philadelphia, PA USA; 36 Oncogenetics Unit, Sheba Medical Center, Tel Hashomer, Sackler Faculty of Medicine, Tel Aviv University, Tel Aviv, Israel; 37 Université Paris 13, Equipe de Recherche en Epidémiologie Nutritionnelle (EREN), Centre de Recherche en Epidémiologie et Statistiques, Institut National de la Santé et de la Recherche Médicale (INSERM U1153), Institut National de la Recherche Agronomique (INRA U1125), Conservatoire National des Arts et Métiers, Communauté d’Université Sorbonne Paris Cité, F-93017 Bobigny, France; 38 Inherited Disease Research Branch, National Human Genome Research Institute, National Institutes of Health, Baltimore, MD USA; 39 Department of Dermatology, Leiden University Medical Centre, Leiden, The Netherlands; 40 Department of Oncology-Pathology, Karolinska Institutet, Karolinska University Hospital, Stockholm, Sweden; 41 Department of Dermatology, Oslo University Hospital, Rikshospitalet, Oslo, Norway; 42 Department of Surgical Oncology, Institute of Oncology Ljubljana, Ljubljana, Slovenia; 43 Department of Surgery, Clinical Sciences, Lund University, Lund, Sweden; 44 Department of Cancer Epidemiology, H. Lee Moffitt Cancer Center and Research Institute, Tampa, FL USA; 45 Department of Genetics, The University of Texas MD Anderson Cancer Center, Houston, TX USA; 46 Department of Medical Genetics, University of Glasgow, Glasgow, UK; 47 McGill University and Genome Quebec Innovation Centre, Montreal, Canada; 48 Department of Public Health, University of Glasgow, Glasgow, UK; 49 Centre for Cancer Research, University of Sydney at Westmead, Millennium Institute for Medical Research and Melanoma Institute Australia, Sydney, Australia; 50 Department of Pathology, Haukeland University Hospital, Bergen, Norway; 51 Department of Molecular Diagnostics, Institute of Oncology Ljubljana, Ljubljana, Slovenia; 52 Department of Oncology/Pathology, Clinical Sciences, Lund University, Lund, Sweden; 53 Melanoma Unit, Dermatology Department & Biochemistry and Molecular Genetics Departments, Hospital Clinic, Institut de Investigacó Biomèdica August Pi Suñe, Universitat de Barcelona, Barcelona, Spain; 54 Inflammatory Bowel Diseases, QIMR Berghofer Medical Research Institute, Brisbane, Australia; 55 Department of Clinical Genetics, Leiden University Medical Center, Leiden, The Netherlands; 56 Cancer Control Group, QIMR Berghofer Medical Research Institute, Brisbane, Australia; 57 Department of Ophthalmology, Flinders University, Adelaide, Australia; 58 Department of Dermatology, University Hospital Essen, Essen, Germany; 59 Menzies Institute for Medical Research, University of Tasmania, Hobart, TAS Australia; 60 Institute of Health and Biomedical Innovation, Queensland University of Technology, Brisbane, QLD Australia; 61 Breakthrough Breast Cancer Research Centre, The Institute of Cancer Research, London, UK; 62 Cancer Epidemiology and Services Research, Sydney School of Public Health, The University of Sydney, Sydney, Australia; 63 Department of Dermatology, Fachklinik Hornheide, Institute for Tumors of the Skin at the University of Münster, Münster, Germany; 64 Department of Community and Family Medicine, Geisel School of Medicine, Dartmouth College, Hanover, NH USA; 65 Sydney School of Public Health and the Melanoma Institute Australia, University of Sydney, Sydney, Australia; 66 Department of Epidemiology and Biostatistics, Memorial Sloan Kettering Cancer Center, New York, USA

Correction to: *Nature Communications*; 10.1038/s41467-018-06649-5; published online: 14 November 2018

The original version of this Article contained an error in the spelling of the authors Fan Liu and M. Arfan Ikram, which were incorrectly given as Fan Lui and Arfan M. Ikram.

Furthermore the original version of this Article contained errors in the author affiliations.

Peter Kraft was incorrectly associated with Department of Epidemiology, Richard M. Fairbanks School of Public Health, Melvin and Bren Simon Cancer Center, Indiana University, Indianapolis, IN 63110, USA.

Fan Liu was incorrectly associated with Department of Epidemiology, Harvard T.H. Chan School of Public Health, Boston 02115 MA, USA.

M. Arfan Ikram was incorrectly associated with Department of Genetic Identification, Erasmus MC, University Medical Centre, Rotterdam, The Netherlands.

Pamela A. Madden, Andrew C. Heath and Elliot C. Nelson were incorrectly associated with Department of Internal Medicine, Erasmus MC, Rotterdam, Netherlands.

Adele C. Green was incorrectly associated with Department of Psychiatry, Washington University School of Medicine, St. Louis, MO 63110, USA.

Stephen Chanock was incorrectly associated with Molecular Oncology Group, CRUK Manchester Institute, University of Manchester, Manchester, UK

Richard A. Sturm was incorrectly associated with Division of Cancer Epidemiology and Genetics, National Cancer Institute, Bethesda, MD, USA.

Manfred Kayser was incorrectly associated with Department of Epidemiology, Harvard T.H. Chan School of Public Health, Boston 02115 MA, USA.

David J. Hunter was incorrectly associated with Department of Epidemiology, Richard M. Fairbanks School of Public Health, Melvin and Bren Simon Cancer Center, Indiana University, Indianapolis, IN 63110, USA

Jiali Han was incorrectly associated with Dermatology Research Centre, University of Queensland Diamantina Institute, Translational Research Institute, Brisbane, Australia

The affiliation of André G. Uitterlinden with Department of Internal Medicine, Erasmus MC, Rotterdam, Netherlands was inadvertently omitted.

Affiliation 5 incorrectly read ‘Department of Dermatology, Erasmus MC, University Medical Centre, Rotterdam, The Netherlands’ and Affiliation 10 incorrectly read ‘Department of Genetic Identification, Erasmus MC, University Medical Centre, Rotterdam, The Netherlands.’

This has now been corrected in both the PDF and HTML versions of the Article.

